# The Important Role of Dendritic Cell (DC) in iNKT-Mediated Modulation of NK Cell Function in Chlamydia pneumoniae Lung Infection

**DOI:** 10.1155/2019/4742634

**Published:** 2019-05-20

**Authors:** Lei Zhao, Xiaoling Gao, Hong Bai, Antony George Joyee, Shuhe Wang, Jie Yang, Weiming Zhao, Xi Yang

**Affiliations:** ^1^Department of Immunology, Faculty of Medicine, University of Manitoba, Winnipeg, Canada; ^2^Institute of Basic Medical Sciences, Qilu Hospital, Shandong University, Jinan, Shandong, China; ^3^Department of Pathogenic Biology, Shandong University School of Medicine, Jinan, Shandong, China

## Abstract

*Chlamydia pneumoniae* (*Cpn*) infection causes multiple acute and chronic human diseases. The role of DCs in host defense against Cpn infection has been well documented. The same is true for invariant natural killer T (iNKT) cells and NK cells, but the interaction among cells is largely unknown. In this study, we investigated the influence and mechanism of iNKT cell on the differentiation and function of NK cell in *Cpn* lung infection and the role played by DCs in this process. We found that expansion of IFN-*γ*-producing NK cells quickly happened after the infection, but this response was altered in iNKT knockout (KO) mice. The expression of activation markers and the production of IFN-*γ* by different NK subsets were significantly lower in KO mice than wild-type (WT) mice. Using in vitro DC-NK coculture and in vivo adoptive transfer approaches, we further examined the role of DCs in iNKT-mediated modulation of NK cell function. We found that NK cells expressed lower levels of activation markers and produced less IFN-*γ* when they were cocultured with DCs from KO mice than WT mice. More importantly, we found that the adoptive transfer of DCs from the KO mice induced less NK cell activation and IFN-*γ* production. The results provided evidence on the modulating effect of iNKT cell on NK cell function, particularly the critical role of DCs in this modulation process. The finding suggests the complexity of cellular interactions in *Cpn* lung infection, which should be considered in designing preventive and therapeutic approaches for diseases and infections.

## 1. Introduction

The role of DCs in host defense against infections has been well defined. In particular, we and others have found significant interaction of iNKT/NK cells with DCs which are important for T cell response in different infection settings [1–4]. In the present study, we intended to study the modulating effect of iNKT cell on NK cell and the involvement of DCs in this process.

NK cells are an important component of the innate immune system, contributing to host resistance to microbial infection such as viruses, bacteria, and certain parasites [5–7]. Some studies have demonstrated that human and murine NK cells are highly heterogeneous populations with multifunctional features [8, 9]. In this context, murine NK cells can be grouped into four subsets based on CD11b and CD27 expression. The CD11b^low^CD27^low^, CD11b^low^CD27^high^, CD11b^high^CD27^high^, and CD11b^high^CD27^low^ NK subsets represent a continuous NK cell differentiation process [10]. Among these subsets, the CD11b^low^CD27^high^ and CD11b^high^CD27^high^ subsets exhibit enhanced cytokine production and higher responsiveness, while CD11b^high^CD27^low^ NK subsets appear to be more tightly controlled due to their higher expression of inhibitory receptors [11]. The functional distinctions of NK subsets in immune responses have been discerned in several disease models [12, 13].

iNKT is an innate-like T lymphocyte sublineage that expresses NK cell markers and limited/semi-invariant T cell repertoire that recognize lipid in the context of nonclassical MHC-I molecule CD1d [14, 15]. Functional studies on iNKT cells have suggested a significant impact of these cells on immune regulation. After activation, iNKT cells produce a broad range of cytokines and provide surface stimulatory molecules to activate NK cells, T cells, B cells, and DCs [1, 16, 17]. Some studies have shown the modulating effect of iNKT cell on NK cell in infection and noninfection settings. Injection of soluble model antigen *α*-galactosylceramide (*α*-Galcer) which is a specific agonist for iNKT cells led to NK cell activation and proliferation and production of IFN-*γ* in mice [18, 19]. In addition, *α*-Galcer-stimulated human iNKT cells also activated NK cells through an IL-2-dependent mechanism [20]. Furthermore, activated iNKT cells induced NK cell differentiation and affected NK cell education [21]. Interestingly, in infection settings, the effect of iNKT on NK cell functions appears variable in the literature [22–24]. One study showed that NK cells maintained its activity and protective function in the absence of iNKT cells during *Trypanosoma cruzi* infection [22]. Another study showed that iNKT cells suppressed IFN-*γ* production by NK cells following acute influenza A virus infection [23]. In contrast, we reported previously that iNKT cells promote IFN-*γ* production by NK cells during *C. muridarum*, a mouse strain of *Chlamydia*, infection [24]. The mixed findings suggest that the effect of iNKT cell on NK cell is likely infection specific; thus, it is important to specifically study particular pathogens.

Members of the *Chlamydia* family are obligate intracellular Gram-negative bacterial pathogens, which include several species and various serotypes. Previous studies have shown that the distinct cellular immune responses including NK/iNKT cells were induced by different species of *Chlamydia* particularly *Cpn* and *C. muridarum* (*Cm*) [25]. NK cells play a protective role in *Cm* infection [26–28], but one report showed that NK cells did not contribute to innate resistance to *Cpn* infection in a setting of T cell and B cell deficiencies [29, 30]. Notably, the kinetics and functional involvement of NK cell response in *Cpn* infection have not been well clarified. Therefore, it is important to specifically test NK cell response and the effect of iNKT cells in *Cpn* infection. In addition, we have reported that iNKT cells can modulate the phenotype, cytokine production, and function of DCs in *Cpn* infection [1, 31]. In particular, we found that iNKT cells could enhance DC IL-12p70 production in a CD40L-, IFN-*γ-*, and cell-cell contact-dependent manner in the coculture system. Considering the important role of DCs in modulation of NK and iNKT cell function, it is natural to study the involvement of DCs in this process.

In the present study, through depletion of NK cell in vivo and direct comparison of WT and iNKT KO mice, we examined the involvement of NK cell response in host defense against Cpn infection and the effect of iNKT cells on NK cell differentiation and activation during the infection. Further, we explored the mechanism by which iNKT cells modulate NK cell activation and function, particularly the involvement of DCs in this process, using in vitro DC-NK coculture and in vivo adoptive transfer approaches.

## 2. Materials and Methods

### 2.1. Mice

Female C57BL/6 mice were bred and kept under a specific pathogen-free animal facility at the University of Manitoba. Breeding pairs of J*α*18 gene knockout (J*α*18-/-) mice which lacked invariant iNKT cells in B6 background were kindly provided by Dr. Masaru Taniguchi (RIKEN Research Center for Allergy and Immunology) and maintained at the animal care facility of the University of Manitoba. All mice used were between 6 and 12 weeks old. All experiments were conducted in accord with the guidelines of the Canadian Council on Animal Care, and the protocol was approved by the ethical committee of the University of Manitoba.

### 2.2. Chlamydia

*Cpn* were propagated, purified, and quantified as previously described [1]. Briefly, *Cpn* was grown in HEp-2 cells in Eagle's MEM containing 10% FBS. After 48 h culture, infected cells were harvested. Elementary bodies (EBs) were purified by discontinuous density gradient centrifugation. The purified EBs were measured by immunostaining and stored at -80°C. For mice infection, 5 × 10^6^ inclusion-forming units (IFUs) of *Cpn* in 40 *μ*l final volume of PBS was used to inoculate mice intranasally. The same seed stock of EBs was used throughout the study.

### 2.3. Antibodies

Fluorescent-labeled mAbs and corresponding isotype controls were purchased from eBioscience or BioLegend. For analysis of NK subsets by flow cytometry, anti-CD3*ε*-PE-Cy7, anti-NK1.1-PE, anti-NK1.1-APC, anti-CD69-PE, anti-CD25-FITC, anti-CD11b-APC, anti-NK1.1-Pacific Blue, anti-CD27-FITC, and anti-CD11b-APC-Cy7 were used.

### 2.4. Surface Marker and Intracellular Cytokine Staining

For *in vivo* experiments, the fresh splenocytes were stained by anti-CD3*ε*-PE-Cy7, anti-NK1.1-APC, anti-CD69-PE, and anti-CD25-FITC for CD69 and CD25 expression. NK subset staining and analysis were performed as described previously [24]. For intracellular cytokine staining, cells were stimulated with PMA (50 ng/ml) and Ionomycin (1 *μ*g/ml) and incubated in complete RPMI 1640 medium at 37°C. After 3-hour incubation, brefeldin A was added, and cells were cultured for another 3 hours to accumulate cytokines intracellularly. Cells were subsequently washed and blocked for 10 min with anti-CD16/CD32 in FACS buffer (Dulbecco's PBS, 2% heat-inactivated FBS, 0.09% sodium azide) and then surface stained with the appropriate Abs. Cells were fixed, permeabilized, and subsequently stained with APC-anti-IFN-*γ* for 30 min. Stained cells were washed and analyzed using an LSR II flow cytometer. The data were subsequently analyzed with FACS express software.

### 2.5. Isolate DCs and Adoptive Transfer

DCs were isolated from splenocytes according to the manufacturer's instructions as described previously [1]. Briefly, spleens from either J*α*18-/- mice or C57BL/6 mice were aseptically collected at a certain time after infection and digested into single cells using 2 mg/ml collagenase D. DCs were purified using magnetic CD11c microbeads and MACS-positive selection column (Miltenyi Biotec, Auburn, CA). The purity of the isolated CD11c+ cells was up to 90% based on flow cytometric analysis.

For adoptive transfer, DCs were isolated from either *Cpn*-infected J*α*18-/- mice or C57BL/6 mice, and 2 × 10^6^ DCs in sterile PBS were adoptively transferred intravenously (i.v.) to naive C57BL/6 recipients. Twenty-four hours after adoptive transfer, the mice were intranasally inoculated with 5 × 10^6^ IFUs of *Cpn*. The recipient mice were sacrificed at day 3 p.i., and the expression of activation markers and IFN-*γ* production by NK cells were measured by flow cytometry.

### 2.6. NK Cell Purification

Spleen cells from uninfected mice were prepared, and NK cells were isolated by negative selection using an NK cell isolation kit (Miltenyi Biotec) according to the manufacturer's instructions. NK cells (NK1.1+CD3e-) were >90% pure following separation as determined by flow cytometry.

### 2.7. DC and NK Cell Coculture

Purified DCs (1 × 10^6^) from either *Cpn*-infected J*α*18-/- mice or C57BL/6 mice were cocultured with NK cells (2 × 10^5^) isolated from uninfected C57BL/6 mice in a 96-well round bottom plate at 37°C. After 48 h, cells were collected for intracellular cytokine staining to analyze IFN-*γ* production by NK cells following the protocol described above. Meanwhile, concentrations of IFN-*γ* in the supernatants were measured by ELISA.

### 2.8. NK Depletion *In Vivo*

Mice were injected i.p. with 30 *μ*l anti-asialo-GM1 (Wako) in 300 ml PBS to deplete NK cells *in vivo*. Control mice received appropriate normal rabbit IgG (Wako). Injections were performed 3 days prior to Chlamydia infection and repeated every 3 days to maintain depletion. The depletion of NK cells (95%) by this treatment was confirmed by flow cytometric analysis.

### 2.9. Statistical Analysis

Statistical analysis of the data was performed as indicated using either unpaired Student's *t*-test or one-way ANOVA (GraphPad Prism software; version 5). Values of *p* < 0.05 were considered significant.

## 3. Results

### 3.1. CD27^high^ NK Cells Expand after Respiratory Infection with *Cpn*

**Figure 1:**
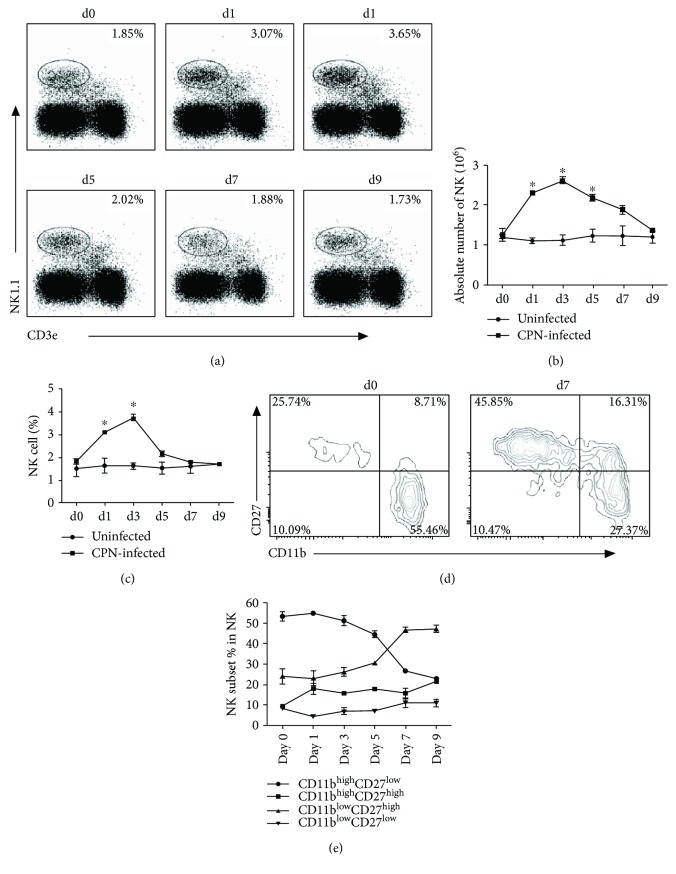
*Cpn* lung infection induces expansion of NK cell number and changes the distribution of NK subsets. Mice were infected with 5 × 10^6^ IFU*Cpn* intranasally and killed at specified time points. Splenocytes were stained for NK1.1 and CD3e. (a) Percentage of NK cells (NK1.1+CD3e-) among living lymphocytes was gated according to forward and side scatter. Representative dot plots are shown. (b) Kinetics of the percentage of NK cells. (c) Kinetics of the absolute number of NK cells per mouse spleen. (d) Based on CD11b and CD27 staining, NK cell subsets were analyzed after gating on NK1.1+CD3e- cells. Representative zebra plots for CD11b- and CD27-expressing NK cells before and after *Cpn* infection were shown. (e) Kinetics of the mean percentage of indicated NK cell subsets are plotted. Data are expressed as mean (*n* = 4) ± SD and represent three independent experiments. ^∗^ *p* < 0.05.

To test whether NK cells respond to *Cpn* infection, the percentage and the absolute number of NK cells were analyzed by flow cytometry at various time points after respiratory tract *Cpn* exposure. NK cells were identified as NK1.1+CD3e- cells. As shown in (Figures 1(a) and 1(b), there was a modest increase of the percentage of NK1.1-expressing cells in the spleen at day 1 after *Cpn* infection which peaked at day 3. At day 5, the percentage of NK cells started to decrease. A similar kinetic change of NK cells was also observed in the lung (Figure S1A and B). The absolute number of NK cells in the spleen also increased at day 1 and reached the peak at day 3 (Figure 1(c)). The total number of NK cells expanded about 2.5-folds in the spleen (Figure 1(c))) and 4-folds in the lung (Figure S1C) at their peak level at day 3.

It has been reported that murine NK cells can be grouped into 4 subsets based on surface CD11b and CD27 expression [10, 11]. To explore whether the NK subsets were affected by *Cpn* infection, the pattern of NK subsets was examined. As shown in Figures 1(d) and 1(e), *Cpn* infection resulted in a significant increase of the proportion of CD27^high^ NK cells compared with uninfected mice. The frequency of both CD11b^low^CD27^high^ and CD11b^high^CD27^high^ NK cells was nearly doubled at day 7 p.i. The increase of CD27^high^ NK cells was accompanied by a reduction of CD11b^high^CD27^low^ NK subset. Together, the results indicate that *Cpn* infection induces rapid expansion of NK cells, especially the CD27^high^ subsets.

### 3.2. NK Cells Are Activated and Produce IFN-*γ* after *Cpn* Infection

**Figure 2:**
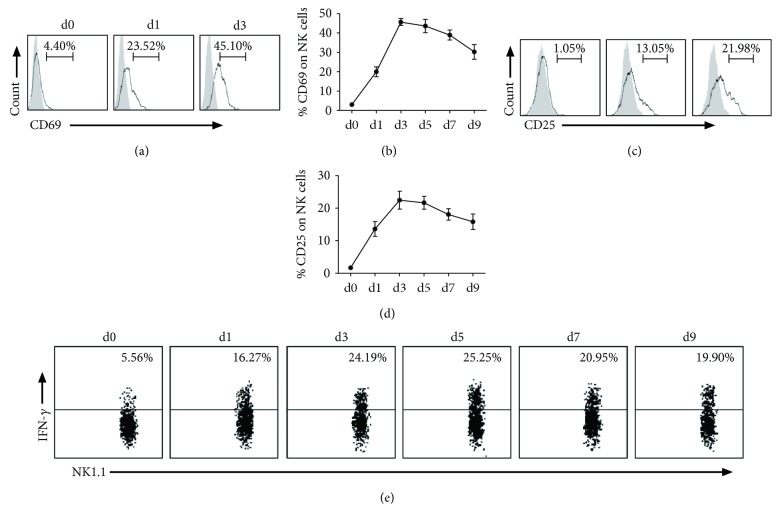
NK cells become activated and produce IFN-*γ* in response to *Cpn* infection. After intranasal infection by *Cpn*, mice were killed at different days, and splenocytes were stained for NK1.1, CD3e, CD25, and CD69. (a) Representative histogram of activation marker CD69 expression on NK cells at days 0, 1, and 3 p.i.: isotype control Ab staining (gray histograms) and infected mice (solid lines). (b) Kinetics of the expression of CD69 by NK cells. (c) Representative graph of the expression of activation marker CD25 on NK cells after infection: isotype control Ab staining (gray histograms) and infected mice (solid lines). (d) Kinetics of the expression of CD25 by NK cells. (e) Representative staining for intracellular IFN-*γ* before and after *Cpn* infection. IFN-*γ* production by splenic NK cells was assayed by intracellular cytokine staining on gated NK1.1+CD3e- population. The data represent one of at least three independent experiments and are shown as mean ± SD for four mice at end time points.

To determine whether NK cells are an activated phenotype in response to *Cpn* infection, we measured the expression of two early activation markers, CD69 and CD25, on NK cells in a kinetic manner. We found that *Cpn* infection rapidly increased CD69 expression on NK cells as early as day 1, peaking at day 3, with 45% NK cells in the spleen (Figures 2(a) and 2(b)) expressing this marker. The level of CD25 on splenic NK cells (Figures 2(c) and 2(d)) was also significantly elevated at day 1 and peaked at day 3 p.i. Both CD69 and CD25 gradually decreased from the peak but remained high at day 9 when the experiment was terminated. Further, intracellular cytokine staining was performed to determine IFN-*γ* production by NK cells following *Cpn* infection. We found that the percentage of IFN-*γ*-producing NK cells started to increase as early as day 1 p.i., with about 25% of NK cells stained positive for IFN-*γ* at day 5 p.i. (Figure 2(e)). About 5% of NK cells produced IFN-*γ* in uninfected control mice. The high IFN-*γ* production by NK cells remained during the period of the study. The results show that NK cells are activated and produce IFN-*γ* in response to *Cpn* infection.

### 3.3. NK Cell Depletion Results in Increased Susceptibility to *Cpn* Lung Infection

**Figure 3:**
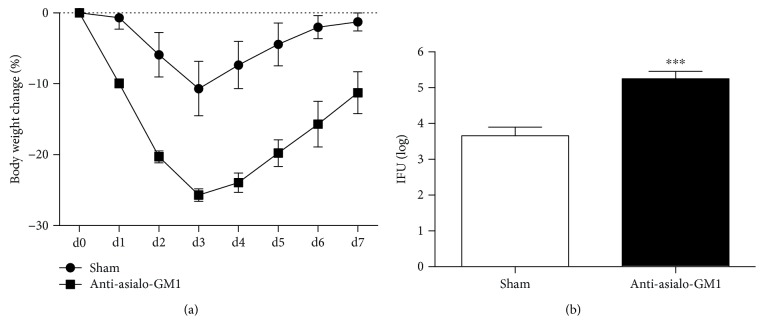
More severe disease in NK-depleted mice after *Cpn* lung infection. C57BL/6 mice were treated with anti-asialo-GM1 or control rabbit IgG during *Cpn* lung infection as described in Materials and Methods. (a) The body weight changes of the mice were monitored daily. Each time point represented the mean ± SD of three mice. (b) Mice were sacrificed, and the lungs were collected for testing *Cpn* loads at day 7 p.i. The mean of log10-transformed IFUs per lung is presented. ^∗∗∗^ *p* < 0.001.

To further confirm the role of NK cells in host defense against *Cpn* lung infection in immune intact mice, we used anti-asialo-GM1 treatment to deplete NK cells in vivo and examine its effect on host susceptibility to *Cpn* infection. We found that NK-depleted mice showed greater body weight loss (Figure 3(a)) and significantly higher bacterial burden in the lung compared with control antibody-treated mice (Figure 3(b)). The depletion of NK cells in the spleen and lung was confirmed by flow cytometry (Figure S2). The result suggested that NK cells played an important protective role in host defense against *Cpn* lung infection.

### 3.4. iNKT Deficiency Leads to Changes of NK Cell Subset Expansion

**Figure 4:**
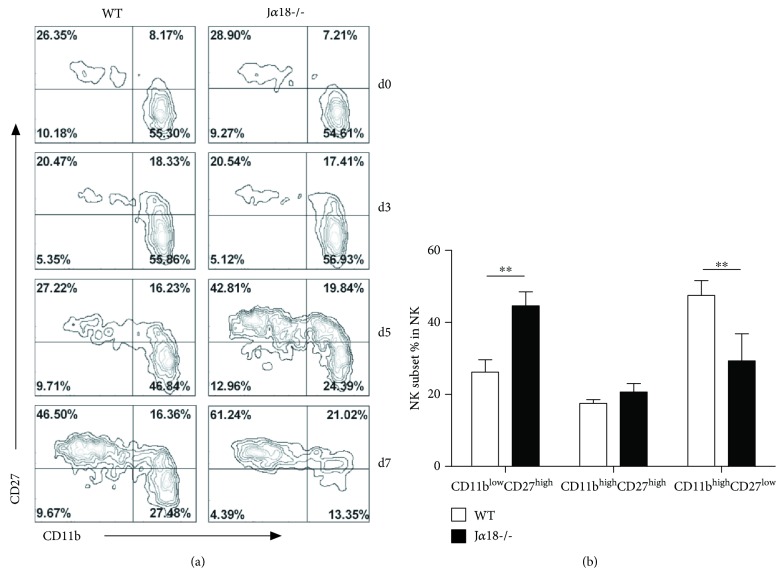
NK cells from J*α*18-/- mice show altered distribution of NK subsets during *Cpn* infection. Mice were killed at different days following intranasal infection with *Cpn* EBs (5 × 10^6^ IFUs), and the splenocytes were prepared for flow cytometry. (a) Relative distribution of CD11b- and CD27-expressing NK cells at a steady state and at specified days after infection in WT mice and J*α*18-/- mice. Zebra plots were shown from at least three independent experiments. (b) The bar summarizes the relative distribution of splenic NK subsets at day 5 p.i., which are shown as mean ± SD for four mice. ^∗∗^ *p* < 0.01.

To address the impact of iNKT cell on the expansion of NK cells during *Cpn* infection, we first measured the number of NK cells in WT and iNKT KO (J*α*18-/-) mice. We found that there is no significant difference in NK cell number between these two mouse strains before and after *Cpn* infection (Figure S3). Then, we analyzed the distribution of NK subsets based on their CD27 and CD11b expression in WT and iNKT KO mice before and after *Cpn* infection. At the steady state before infection, the distribution of NK subsets was similar in iNKT KO and WT control mice. Following *Cpn* infection, the pattern of NK subsets was significantly altered in WT mice at day 7 p.i., showing a remarkable increase of the CD11b^low^CD27^high^ and CD11b^high^CD27^high^ NK subsets (Figure 4). Notably, the increase of the CD27^high^ NK cells in iNKT KO mice was found at day 5 p.i., earlier than that shown in WT mice. Consequently, the frequency of CD11b^high^CD27^low^ NK subset showed an earlier decrease in iNKT KO mice than WT control. Therefore, iNKT deficiency appeared to promote the increase of CD27^high^ NK subset in the kinetics.

### 3.5. iNKT Deficiency Results in Compromised NK Activation and Reduced IFN-*γ* Production by NK Cells following *Cpn* Infection

**Figure 5:**
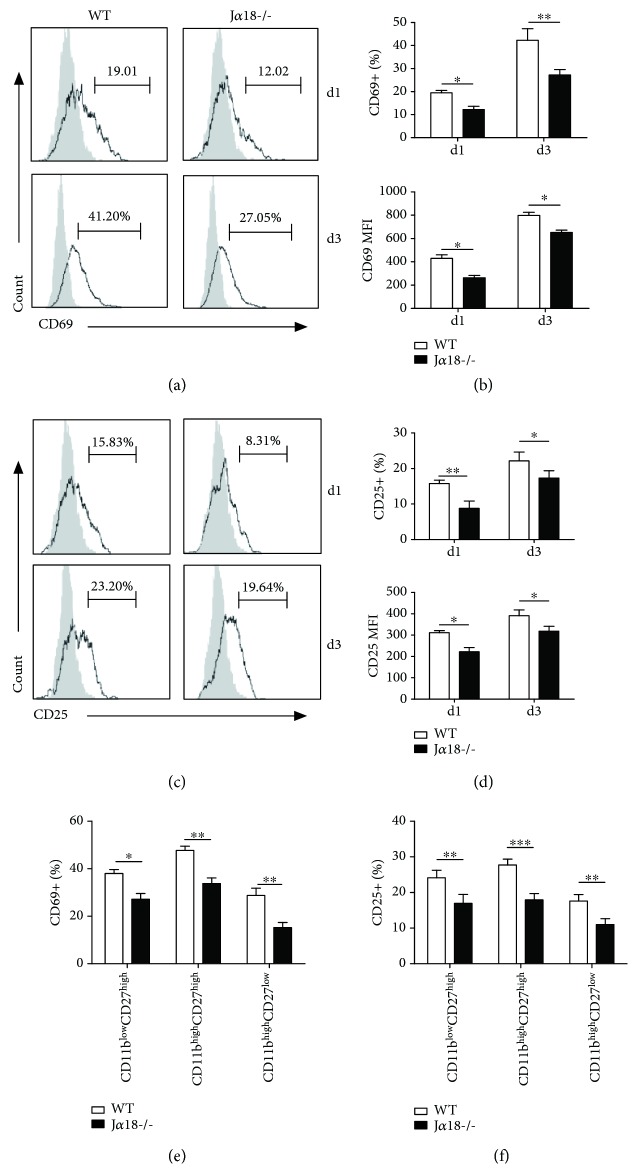
NK cells from J*α*18-/- mice show lower CD69 and CD25 expression in response to *Cpn* infection. WT and J*α*18-/- mice were intranasally infected with *Cpn* (5 × 10^6^ IFUs) and examined at days 1 and 3 p.i. Splenocytes were prepared for flow cytometry analysis. (a) CD69 expression on NK (NK1.1+CD3e-) cells was analyzed at days 1 and 3 p.i. Representative histograms are shown. Filled gray: isotype control Ab staining; black lines: anti-CD69 Ab staining. (b) Summary of the proportion (upper) and the MFI (lower) of CD69 on NK cells. (c) Representative histograms of CD25 expression on NK cells are shown. Filled gray: isotype control Ab staining; black lines: anti-CD25 Ab staining. (d) Summary of the proportion (upper) and the MFI (lower) of CD25 on NK cells. (e) The frequency of CD69 was analyzed on the specified NK cell subsets based on CD11b and CD27 staining at day 3 p.i. (f) The frequency of CD25 was analyzed on the indicated NK cell subsets based on CD11b and CD27 staining at day 3 p.i. The results are shown as mean ± SD of three mice in each group and are representative of three independent experiments. ^∗^ *p* < 0.05 and ^∗∗^ *p* < 0.01.

It has been reported that the murine CD27^high^ NK cells exhibit potent responsiveness and enhanced cytokine production, whereas the CD11b^high^CD27^low^ subset has a higher activation threshold [11]. Therefore, the increased frequency of the CD27^high^ NK cells in J*α*18-/- mice might suggest an enhanced NK function in these mice. To test this possibility, we compared the activation status and cytokine production of NK cell in the *Cpn*-infected WT and iNKT KO mice. Surprisingly, we found that the NK cells from iNKT KO mice showed much lower expression of CD69 (Figures 5(a) and 5(b)) and CD25 (Figures 5(c) and 5(d)) than WT mice at day 1 and day 3 p.i. Further analysis revealed that the levels of CD69 (Figure 5(e)) and CD25 (Figure 5(f)) expression in all the NK subsets from iNKT KO mice were lower than that of WT mice. The data did not support the notion of higher NK activation in iNKT KO mice than WT mice although the percentage of CD27^high^ NK cells is higher in iNKT KO mice.

**Figure 6:**
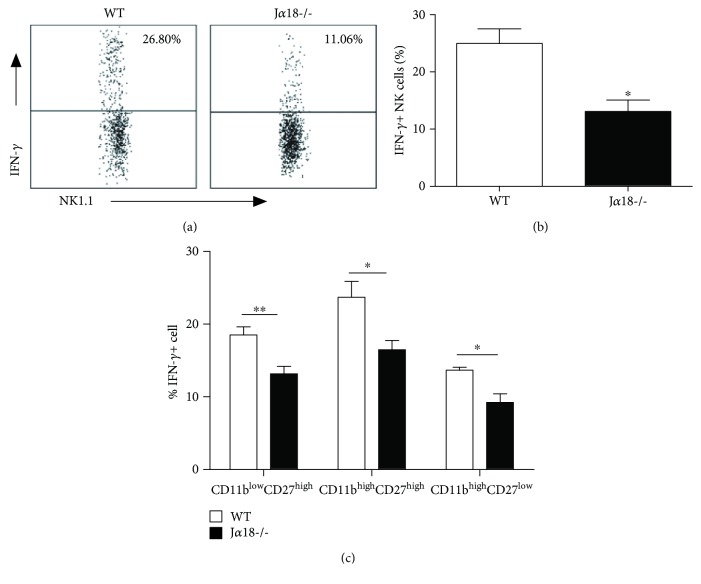
Reduced IFN-*γ* production by NK cells in J*α*18-/- mice following *Cpn* lung infection. Mice were intranasally infected with *Cpn* (5 × 10^6^ IFU). At day 5 p.i., splenocytes from WT and J*α*18-/- mice were stained for NK1.1, CD3e, CD11b, CD27, and IFN-*γ* and analyzed by flow cytometry. (a) Representative dot plots of IFN-*γ* production by NK cells in WT and J*α*18-/- mice. (b) The percentage of IFN-*γ*-producing NK cells was summarized. (c) The frequency of IFN-*γ* production was analyzed on the specified NK cell subsets based on CD11b and CD27 staining. The data are shown as mean ± SD (*n* = 4). One representative experiment of three independent experiments is shown. ^∗^ *p* < 0.05.

We further investigated the influence of iNKT cells on IFN-*γ* production by NK cells through intracellular cytokine staining. As shown in Figures 6(a) and 6(b), significantly less NK cells in iNKT KO mice were stained positively for IFN-*γ* at day 5 after *Cpn* infection, compared with those in WT mice. The percentage of IFN-*γ*-producing NK cells in iNKT KO mice was less than half of WT mice. IFN-*γ* production by NK cells partially depended on iNKT cells. Furthermore, we tested the levels of intracellular IFN-*γ* expression in each of the subsets to determine the functional changes in individual NK subsets. We found reduced IFN-*γ* production by all the three major NK subsets (Figure 6(c)), suggesting that the modulating effect of iNKT cells on IFN-*γ* production by NK cells was not restricted to particular NK subsets. Together, the data suggest that iNKT cells can promote the activation and IFN-*γ* production of NK cells following *Cpn* infection.

### 3.6. DCs from iNKT-Deficient Mice Show Reduced Ability to Induce NK Cell Activation and IFN-*γ* Production

**Figure 7:**
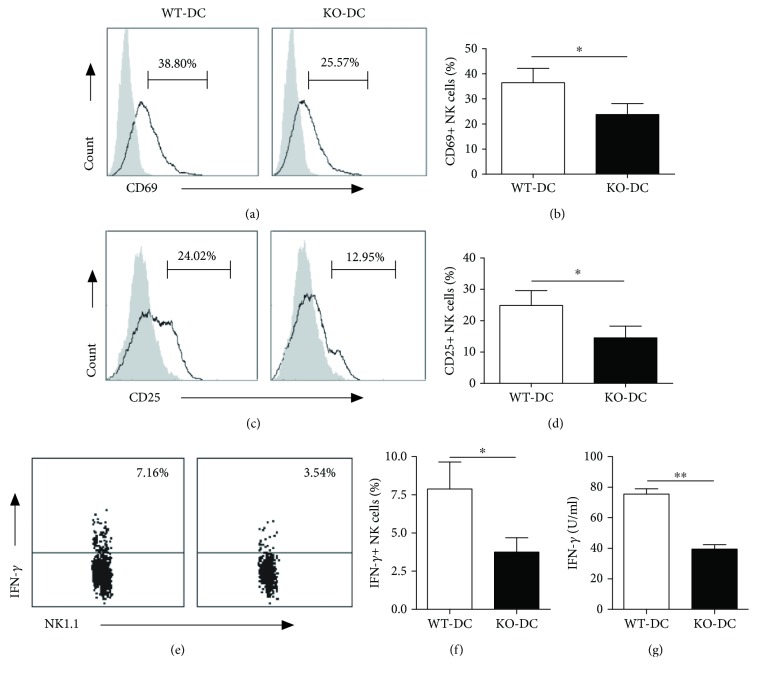
Reduced ability of DCs from *Cpn*-infected iNKT KO mice to induce activation and IFN-*γ* production by naïve NK cells. DCs were isolated from spleens of iNKT KO mice (KO-DC) and WT mice (WT-DC) at day 5 p.i. NK cells were enriched from the spleen of naïve mice. NK cells (2 × 10^5^) were cocultured with DC (1 × 10^6^) cells in a 96-well round bottom plate. After 48 h, the cells and supernatant were collected for CD69, CD25, and IFN-*γ* staining of the cells and ELISA measurement of IFN-*γ* in the supernatants, respectively. The representative histograms and dot plots for CD69 (a) and CD25 (b) expression and IFN-*γ* production (e) by NK cells are depicted. The percentage of CD69+ (b), CD25+ (d), and IFN-*γ*+ (f) NK cells was summarized. (g) The concentration of IFN-*γ* in supernatant was examined by ELISA. The data represent one of three similar experiments and are shown as mean ± SD. ^∗^ *p* < 0.05, ^∗∗^ *p* < 0.01.

**Figure 8:**
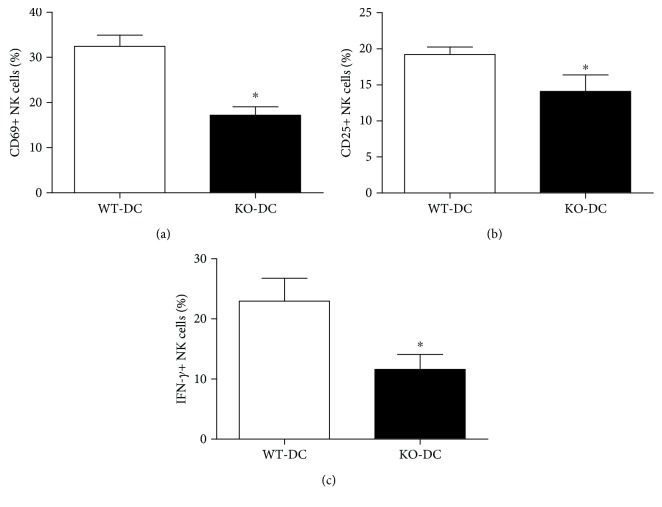
Adoptive transfer of KO-DC induces less activation and IFN-*γ* production by NK cells. Naive recipient mice (C57BL/6) were delivered DCs isolated from spleens of *Cpn*-infected iNKT KO mice (KO-DC) and WT mice (WT-DC). The recipient mice were sacrificed at day 3 after *Cpn* challenge. The spleen cells were collected to measure CD69 and CD25 expression and IFN-*γ* production by NK cells using flow cytometry. The percentage of CD69+ (a), CD25+ (b), and IFN-*γ*+ (c) NK cells was summarized. The data represent one of three similar experiments and are shown as mean ± SD. ^∗^ *p* < 0.05.

DC can prime resting NK cell both in mice [4, 32, 33] and in humans [34]. Previous studies have demonstrated a pivotal role for DC-derived IL-12 in the induction of IFN-*γ*-producing NK cells [35]. The level of DC maturation affected the degree of NK activation [33, 36]. Therefore, it is likely that the reduced NK cell activation and IFN-*γ* production in iNKT-deficient mice are a result of the altered DC function. To directly test this, DCs were isolated from *Cpn*-infected WT and iNKT KO mice and cocultured with NK cells from uninfected WT mice for 24 hours. The expression of CD69 and CD25 and production of IFN-*γ* by NK cells were tested by flow cytometry. As shown in Figure 7, both WT-DC and KO-DC induced enhanced CD69 and CD25 surface expression by NK cells. However, KO-DC-induced CD69 (Figures 7(a) and 7(b)) and CD25 (Figures 7(c) and 7(d)) expression of NK cells was significantly lower than that induced by WT-DC. More importantly, NK cells cocultured with KO-DC produced significantly lower levels of IFN-*γ* than those cocultured with WT-DC (Figures 7(e) and 7(f)). Meanwhile, IFN-*γ* levels in culture supernatants were also significantly lower in KO-DC-NK coculture than in WT-DC-NK coculture (Figure 7(g)). As a control, neither DC nor NK alone produced detectable IFN-*γ*. To further test the role of DCs in iNKT-mediated NK response in vivo, we performed adoptive transfer experiments with DC isolated from *Cpn*-infected iNKT KO and WT mice. The activation marker expression and IFN-*γ* production by NK cells were examined at day 3 after *Cpn* challenge. As shown in Figure 8, significantly lower CD69 and CD25 expression and IFN-*γ* production by NK cells were observed in the recipients of KO-DCs than WT-DC recipients. The data suggest that DCs play a critical role in iNKT-mediated NK activation and IFN-*γ* production during *Cpn* infection.

## 4. Discussion

Our data showed a significant modulating effect of iNKT cell on NK cell activation and IFN-*γ* production in lung *Cpn* infection. Moreover, we found that DCs play an important role in the iNKT-mediated modulation of NK cell function. Specifically, we showed that respiratory *Cpn* infection induced NK cell activation and IFN-*γ* production with dynamic changes of NK subsets. Further comparison of NK cell response in iNKT KO and WT mice revealed a promoting role of iNKT cells in NK cell function, which was demonstrated by decreased expression of activation markers, reduced production of IFN-*γ*, and changes of NK subsets in iNKT KO mice. The activation and IFN-*γ* production by NK cells are largely influenced by iNKT cells in *Cpn* infection although it is not completely dependent on iNKT cells, suggesting a promoting role of iNKT cell on NK cell function. More interestingly, we found in a DC-NK coculture system that DCs isolated from *Cpn*-infected iNKT KO mice induced lower activation marker expression and less IFN-*γ* production by NK cells than those isolated from WT mice, suggesting a critical role of DCs in iNKT-mediated modulation of NK function. We also showed in the present study that NK cells play an important protective role in host defense against *Cpn* infection. In line with this, we also found that adoptive transfer of DCs from iNKT KO mice generated less IFN-*γ* production by NK cells than the DCs from WT mice in vivo. Our previous studies focus on the effect of iNKT on DC function in modulating T cells [1] while the current data showed an effect of iNKT on DCs which subsequently modulate NK cell function. We have reported previously that iNKT can significantly influence DC phenotype, cytokine production, subsets, and function in the spleen and lung in chlamydial infections [1, 27, 28, 31]. The combined data from previous reports and the current study provide a mechanistic explanation for the previously reported modulating effect of iNKT on NK function [24] by showing the critical role of DCs in this modulating process. Considering our previous report on the modulating effect of NK on DCs [27], the findings also suggest a reciprocal influence of DC and NK cells in chlamydial infection.

The study provides new data on the dynamic changes of NK cell responses in lung *Cpn* infection, showing that NK cells rapidly expanded with upregulated CD69 and CD25 expression and enhanced IFN-*γ* production. More interestingly, the data provided in vivo evidence of NK subset switch in response to *Cpn* infection. Distinct function of NK subsets based on CD27 and CD11b expression has been reported [12, 37, 38]. We observed that *Cpn* infection increased the percentage of CD11b^low^CD27^high^ and CD11b^high^CD27^high^ NK subsets so consequently reducing the proportion of CD11b^high^CD27^low^ NK subset. It has been reported that CD27^high^ NK cells have a lower threshold for activation and produce more IFN-*γ* compared with CD27^low^ subset [11]. In our infection model, CD27^high^ NK cells were the major producer for IFN-*γ* within the whole NK population during *Cpn* infection (data not shown). The increased proportion of CD27^high^ NK cells might contribute to high levels of IFN-*γ* production by NK cells after *Cpn* infection. Although the exact mechanism accounting for CD27^high^ NK cell expansion remains unclear, the following possibilities are likely involved: (1) the proliferation of CD27^high^ NK cells *in situ*, (2) the migration of immature NK cells from bone marrow, and (3) the cell death or reversion of CD11b^high^CD27^low^ NK subset.

The effect of iNKT on NK subsets was particularly interesting. It was found that the iNKT KO mice showed higher expansion of CD27^high^ NK subset than WT mice following *Cpn* infection. Considering the lower threshold of this subset for activation, one might think that the KO mice had better NK function especially IFN-*γ* production. However, we found that all the NK cell subsets including the CD27^high^ subset in iNKT KO mice showed lower CD69 and CD25 expression and IFN-*γ* production compared with the corresponding subsets in WT mice. The results suggest that the surface markers in defying the functional subsets of NK cells may not be absolute. Although CD27 expression normally suggests higher activation and IFN-*γ* production, the CD27^high^ NK cells, in the condition of iNKT deficiency, actually expressed lower levels of activation markers and IFN-*γ* than those in WT mice. Therefore, the analysis of cell subsets needs to have a combination of surface markers and cytokine patterns. Moreover, since all the three NK subsets from iNKT KO mice showed lower expression of activation markers and production of IFN-*γ*, the modulating effect of iNKT cells on NK cells is not restricted to a particular NK cell subset.

The results of NK-DC coculture study provide new mechanistic data on the modulating effect of iNKT on NK cells by showing the critical role of DCs in this process. Notably, we and others have shown the modulating effect of iNKT and NK cells on DC function [1, 27, 28]. In particular, we found that iNKT as well as NK cell can modulate the function of splenic and pulmonary DCs in promoting CD4 and CD8 T cell responses [27, 28]. The present finding on the promoting effect of DCs from iNKT intact WT mice on NK cells for the production of IFN-*γ* suggests a loop of positive feedback in amplifying the promoting role of iNKT on type 1 T cell responses in addition to the direct promoting effect on NK effector function. The finding further supports the importance of interactions among multiple types of innate and adaptive immune cells in the expansion and restriction of the scales of inflammatory reactions for immune homeostasis.

Our data showed that NK depletion in immune intact mice led to increased *Cpn* burden in the lung (Figure 3), suggesting that NK cells are functionally involved in protection against *Cpn* lung infection. This observation might be considered inconsistent with the study using T cell- and B cell-deficient mice [29, 30]. Indeed, one report showed that RAG-1^−/−^/*γ*cR^−/−^ and RAG-1^−/−^ mice showed similar susceptibility to *Cpn* lung infection, suggesting a negligible role of NK cells in *Cpn* infection [30]. Another report showed that the *Cpn* load in the lung of RAG-1^−/−^ mice was not altered after eliminating NK cells using neutralizing Abs [29]. However, we think that our data are not necessarily contradictory with the previous reports. The observed discrepancy might be due to the different mice that were used in the studies: T/B cell deficient [29, 30] *versus* T/B intact mice in the present study. When our current study is considered with previously reported ones [29, 30], it might suggest that NK cell plays its protective role though promoting the function of other immune cells, particularly T and B cells, rather than directly controlling *Cpn* lung infection as an effector. The deficiency of T and B cells in RAG-1^−/−^ mice likely covered up this promoting function of NK cells on Th1 responses though IFN-*γ* production as shown in our previous study [27].

## 5. Conclusions

In summary, our data revealed a significant modulating effect of iNKT cell on NK cell activation and IFN-*γ* production in respiratory *Cpn* infection. Additionally, the results provided a mechanistic explanation for the iNKT-mediated modulation of NK cell function by showing the important role of DCs. The finding has implications on understanding the complexity of cellular networks in respiratory tract chlamydial infection, which should be considered in designing preventive and therapeutic approaches.

## Data Availability

The data used to support the findings of this study are available from the corresponding author upon request.

## Supplementary Materials

Supplementary Materials Figure S1: pulmonary NK cells expand after Cpn lung infection. Figure S2: NK cell depletion in the spleen and lung. Figure S3: the absolute number of NK cells in WT and iNKT KO mice is similar before and after Cpn infection.
