# Supplementary material for: The Important Role of Dendritic Cell (DC) in iNKT-Mediated Modulation of NK Cell Function in Chlamydia pneumoniae Lung Infection
Source: Mediators Inflamm. 2019 May 20;2019:4742634. doi: 10.1155/2019/4742634 (PMC6545808; doi:10.1155/2019/4742634)
Supplement: Supplementary Materials — Figure S1: pulmonary NK cells expand after Cpn lung infection. Figure S2: NK cell depletion in the spleen and lung. Figure S3: the absolute number of NK cells in WT and iNKT KO mice is similar before and after Cpn infection. [file 4742634.f1.pdf]

Supporting information

S1. Pulmonary NK cells expand after *Cpn* lung infection.

S2. NK-cell depletion in the spleen and lung.

S3. The absolute number of NK cells in WT and iNKT KO mice is similar before and after *Cpn* infection.

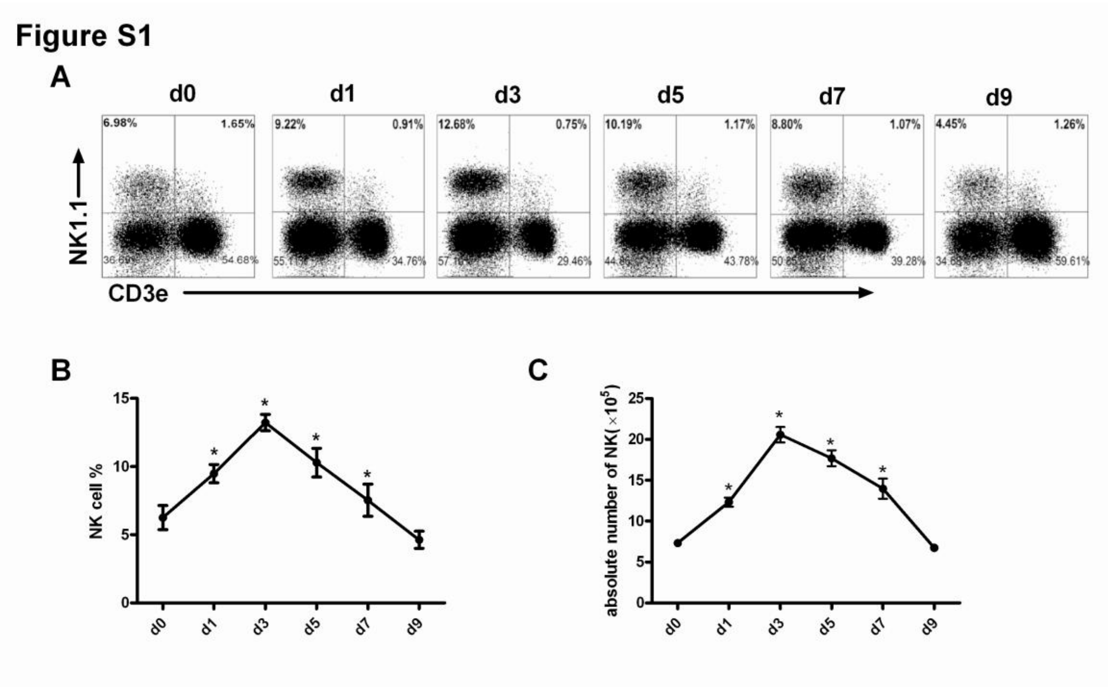

Figure S1. Pulmonary NK cells expand after *Cpn* lung infection. Mice were infected with 5×10^6IFU *Cpn* intranasally and killed at specific time points. Lung mononuclear cells were prepared as described in *materials and methods* and stained for NK1.1 and CD3e. (A) Percentage of NK cells. Representative dot plot are shown. (B) Kinetics of the percentage of NK cells. (C) Kinetics of the absolute number of NK cells per mouse lung. Data are expressed as mean(n=3)±SD and represent three independent experiments. *$p<0.05$.

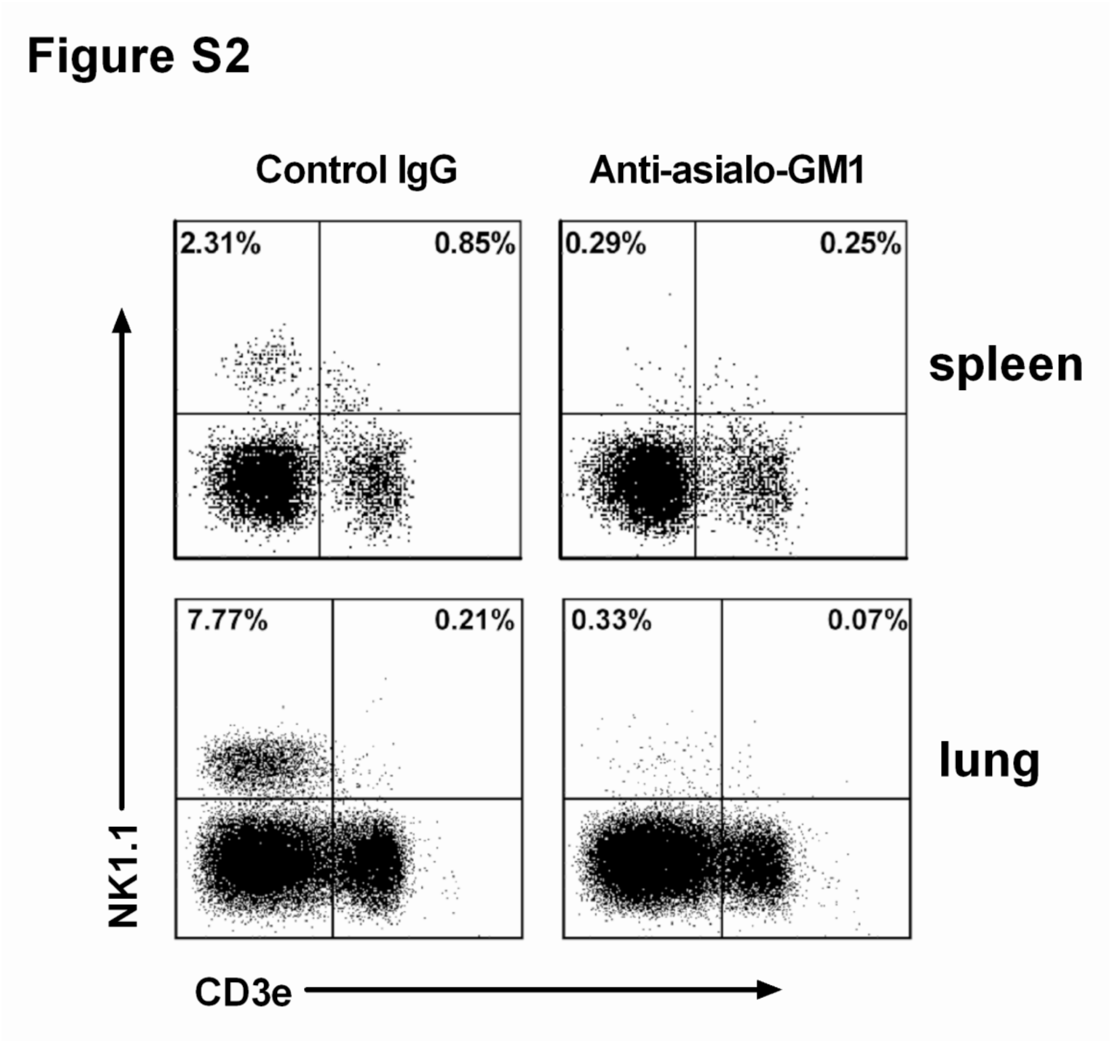

Figure S2. NK-cell depletion in the spleen and lung. NK-cell depletion were done by antiasialo-GM1 antibody treatment. At day 7 p.i., NK1.1+CD3e- cells were gated and identified as NK cells by flow cytometry. Flow cytometric plots showing NK-cell depletion in the spleen and lung.

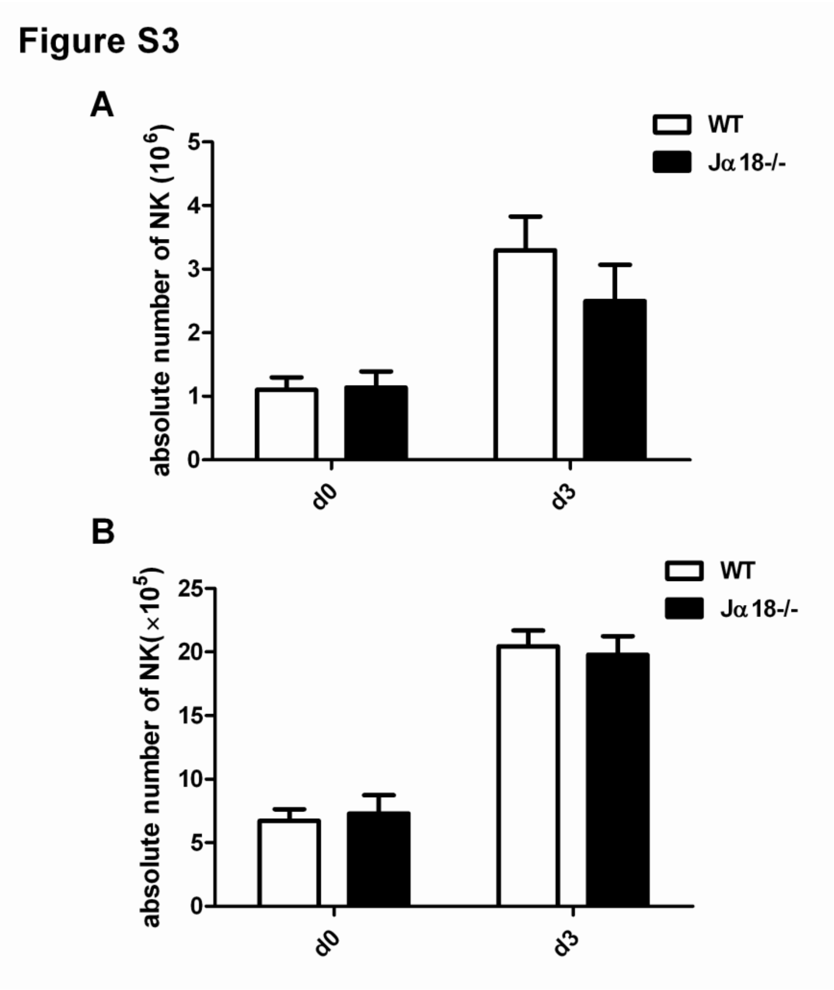

Figure S3. The absolute number of NK cells in WT and iNKT KO mice is similar before and after *Cpn* infection. (A) The absolute number of NK cells per spleen at day 0 and day 3 p.i.. (B) The absolute number of NK cells per lung at day 0 and day 3 p.i.. Data are expressed as mean(n=3)±SD and represent three independent experiments.
